# Plant Proteomics: New Insights Into the Green World Through Advanced Mass Spectrometry

**DOI:** 10.1016/j.mcpro.2025.101070

**Published:** 2025-10-13

**Authors:** Zhi-Yong Wang, Michael R. Sussman

**Affiliations:** 1 Department of Plant Biology, Carnegie Institution for Science, Stanford, California, USA; 2 Center for Genomic Science Innovation, University of Wisconsin Madison, Madison, Wisconsin, USA; 3 Department of Biochemistry, University of Wisconsin Madison, Madison, Wisconsin, USA

Through photosynthesis, plants turn carbon dioxide and water into sugars and oxygen and thus, are not only the ultimate sources of our food and medicine but also fundamental for the health of our global ecosystems and for climate stability. Improving the productivity and resilience of plants is crucial for addressing the food and climate challenges that the world is facing. As sessile organisms without a nervous system, plants must respond and adapt to environmental changes by altering their physiological and developmental programs and have evolved unique molecular mechanisms of intracellular signal transduction that provide high levels of environmental responsiveness and developmental plasticity. The molecular mechanisms underlying plant growth and acclimation are believed to be complex and intricate, and their study benefits greatly from the application of the latest proteomic technologies. This special issue of *Molecular and Cellular Proteomics* presents a wide range of proteomic studies in plants, highlighting both advances in methodologies and insights into biology, particularly plant responses to various internal and external signals, including sugar, nitrogen, hormones, touch, pH, high light, salt, and osmotic stresses.

## Technical Advances

Optimization of sample preparation and instrument operation is critical for efficient proteomic analysis of plant samples, which tend to be chemically and proteomically complex. Several articles in this special issue report improvements in methodologies. Rodriguez Gallo *et al*. (1) introduced a high-throughput quantitative proteomic workflow, multi–compensation voltage (Multi-CV) FAIMSpro BoxCar data-independent acquisition (DIA), which combines multi-CVCV, FAIMSpro (high-field asymmetric waveform ion mobility spectrometry), and BoxCar DIA to achieve optimal balance of throughput and data coverage.

Sample enrichment is key for proteomic analysis of post-translational modifications. Chen *et al*. (2) introduced a novel tandem enrichment strategy, termed TIMAHAC, for tandem suspension trap (S-Trapping), immobilized metal ion affinity chromatography, and hydrophilic interaction chromatography, which allows simultaneous analysis of phosphoproteomes and N-glycoproteomes in the same samples. The metabolic glycan labeling strategy was used for profiling the N-glycoproteome in rice (3). The coverage and confidence of the O-GlcNAcylated proteome were improved by combining wheat germ lectin-weak affinity chromatography enrichment with high-pH reverse-phase fractionation and quantitative proteomic comparison between wildtype and the *ogt* mutant (4). Proteomic profiling of N-terminal acetylation and lysine acetylation was applied in studies of plastid protein acetylation during high-light stress responses (5, 6).

Protein–protein interaction (PPI) is the basis of signal transduction and cellular regulation. PPI can be analyzed by mass spectrometry (MS) following immunoprecipitation (IP-MS, also called affinity purification–MS (AP–MS) or affinity enrichment–MS), *in vivo* proximity labeling (PL-MS), and chemical crosslinking–MS (XL–MS). TurboID-based proximity biotin labeling–MS (TbPL–MS) is considered more sensitive than IP–MS and XL–MS, particularly for detecting transient PPIs such as those mediating post-translational modifications. A kinase-TurboID fusion protein transiently and catalytically modifies the substrate proteins with both phosphate and biotin, enabling biotin-based high-affinity purification and identification of *in vivo* kinase substrates (7, 8). TbPL-MS was applied in several studies to map signaling pathways, such as nutrient sensing (9) and touch responses (10, 11). It was noted that only a limited overlap was found between AP–MS and TbPL–MS datasets of the same bait protein, suggesting the two approaches are perhaps complementary (9). To quantify specificity in TbPL-MS data, the biotin occupancy ratio was calculated as a measure of biotinylation efficiency, which correlates with the distance to the bait protein and thus relative specificity (11). In contrast to TbPL, pupylation (PUP)-based interaction tagging labels proteins that interact directly with the protein fused to the PUP ligase (12). Lin *et al.* (13) used PUP-interaction tagging–MS to identify interactors of a receptor kinase. Another complementary approach to identifying kinase substrates is *in vitro* phosphorylation of synthetic peptides followed by MS analysis, named the kinase client assay (14).

## Biological and Mechanistic Insights

Two articles from Ning Li’s team report proteomic and functional studies of plant touch responses, also known as thigmomorphogenesis. Plants respond to mechanical forces, including external forces, such as wind and touch, as well as internal forces from differential growth of neighboring cells. Repeated touch stimulation reduces growth and delays flowering in *Arabidopsis*. A previous proteomic study by Ning Li’s laboratory identified mitogen-activated protein kinase kinases (MKK1 and MKK2) and the WEB1/PMI2-related protein WPRa4 (TREPH1) as touch-responsive phosphoproteins in *Arabidopsis* (15). In two follow-up studies, TbPL–MS and XL–MS analyses were performed to identify proteins associated with MKK1, MKK2, and WPRa4 (10, 11). The identified proximal proteins of MKK1 and MKK2 include RAF36 kinase, which was further shown to function in touch-induced gene expression and PATL3 phosphorylation, confirming RAF36 function in mechanosignal transduction pathways mediating both wind mechanoresponse and gravitropism (10). The TbPL–MS/XL–MS study led to the discovery of the essential function of the plastoskeleton protein Plastid Movement-Impaired 4 (PMI4, also known as FtsZ1) in touch responses. The findings support a model where the interconnected cytoskeleton–plastoskeleton networks function as a mechanosensory system, acting upstream of the RAF36–MKK1/2 mitogen-activated protein kinase module (11).

Mechanical damage causes leakage of cytoplasmic content, including ATP, which triggers cellular responses through the ATP receptor kinase P2K1 (14). Kinase client-MS screening using synthetic peptide libraries identified hundreds of peptides phosphorylated by the P2K1 kinase *in vitro*. These include several known P2K1 substrates reported previously and new P2K1 substrates involved in extracellular ATP signaling (14).

The perception of microbe-associated molecular patterns by pattern recognition receptors at the host cell surface triggers the first line of defense against microbial pathogens. Watkins *et al*. (16) reported that the bacterial microbe–associated molecular pattern flagellin peptide flg22 triggers a rapid (in 3 min) and massive change in protein abundance and phosphorylation state of over 2000 proteins in the *Arabidopsis* roots, and strikingly, over 95% of these changes were absent in a mutant deficient in heterotrimeric G-protein–coupled signaling. The fungal disease Fusarium head blight causes not only yield loss but also contamination of grains with harmful mycotoxins. Buchanan *et al*. (17) studied the wheat proteome upon exposure to a common mycotoxin, deoxynivalenol. The study identified putative mycotoxin-detoxifying proteins, suggesting a new avenue for identifying detoxifying proteins, which serve as biomarkers for selected breeding strategies.

Salt, drought, and osmotic stresses significantly reduce crop yield worldwide. Rodriguez Gallo *et al*. reported a detailed time-course study of the proteomic responses induced by exposure to osmotic (300 mM mannitol) and salt (150 mM NaCl) stresses. Using the optimized short gradient multi-CV FAIMSpro BoxCar DIA workflow, the study identified salt- and/or osmotic-responsive proteins among nearly 10,000 total uniquely quantified protein groups in roots and shoots. The deep proteomic coverage of temporal response kinetics reveals overlapping and unique responses in protein abundance between osmotic and salt stresses in shoot and root tissues (1). Osmotic stress is known to cause rapid calcium influx and activation of RAF kinases and SnRK2 kinase cascades. Sang *et al*. (18) investigated the proteomic effects of EGTA depleting extracellular calcium, using DIA-based phosphoproteomics, revealing a substantial overlap in protein phosphorylation events triggered by EGTA and hyperosmolarity. The effects of rhizospheric pH on the proteome and phosphoproteome of shoot and root tissues were studied using tandem mass tag-labeling MS, leading to the identification of pH-responsive proteins with functions in root growth under aberrant pH conditions (19).

High light intensity causes stress and damage, particularly to the photosynthetic machinery. Eirich *et al*. (5) investigated the role of plastid acetyltransferase GNAT2 in plant acclimation to short-term light changes. The work suggests that plants use distinct acclimation strategies, involving GNAT2-mediated lysine acetylation, but not N-terminal acetylation, for regulating state transitions of the photosynthetic antenna proteins in rapid environmental responses. The research team further shows that the closely related homolog GNAT1 forms a complex with GNAT2 and that they share N-terminal acetylation substrate sites *in vivo* (6).

Nitrogen is a primary driver of crop yield in agriculture. The interplay between carbon/nitrogen metabolism is crucial for optimal plant growth and involves the protein kinases Sucrose Nonfermenting1-Related Kinase 1 (SnRK1) and Target Of Rapamycin (TOR), two ancient central metabolic regulators. To identify N-dependent interactors, Persyn *et al.* used AP–MS and TbPL–MS to generate comprehensive SnRK1 and TOR interactomes in *Arabidopsis* cell cultures during N-starved and N-repleted growth conditions, identifying a large number of N-dependent interactors with potential functions in the crosstalk of carbon/nitrogen signaling (9).

Plants use hundreds of receptor kinases to sense and respond to diverse chemical signals. The *Arabidopsis* genome encodes over 220 leucine-rich repeat receptor–like kinases, including the brassinosteroid receptor BRI1 and sugar-activated SIRK1. To understand the molecular mechanisms of signaling specificity, Xi *et al.* performed AP–MS analysis of interactomes of domain-swapped chimeras of SIRK1 and BRI1. The findings attribute the extracellular domain, transmembrane domain, juxtamembrane domain, and kinase domain of the respective ligand-binding receptors to their interaction with their coreceptors and substrates (20).

Protein glycosylation plays key roles in signaling. Using TIMAHAC, Chen *et al.* (2) studied the simultaneous changes in protein phosphorylation and N-glycosylation in response to ABA, a stress hormone for abiotic stresses. The study suggests that the crosstalk between phosphorylation and N-glycosylation plays a role in ABA responses (2). About 673 N-glycosylated proteins were identified in rice (3), O-glycosylation of nucleocytoplasmic proteins, including O-GlcNAc and O-fucose modifications, plays crucial roles in nutrient sensing and cellular signaling (3, 21). Improved methodology resulted in the identification of a total of 489 O-GlcNAc-modified proteins in *Arabidopsis* (4). Future functional study of these glycosylation events will shed light on the glycosylation-mediated regulatory pathways and the cellular network integrating O-glycosylation and phosphorylation pathways (21). The application of advanced MS technologies and improved proteomic methodologies in plant research will continue to yield exciting discoveries and deepen our understanding of the green organisms that provide us with food, medicine, and a livable environment.
